# Disruption of Parenting Behaviors in California Mice, a Monogamous Rodent Species, by Endocrine Disrupting Chemicals

**DOI:** 10.1371/journal.pone.0126284

**Published:** 2015-06-03

**Authors:** Sarah A. Johnson, Angela B. Javurek, Michele S. Painter, Michael P. Peritore, Mark R. Ellersieck, R. Michael Roberts, Cheryl S. Rosenfeld

**Affiliations:** 1 Bond Life Sciences Center, University of Missouri, Columbia, MO, 65211, United States of America; 2 Biomedical Sciences, University of Missouri, Columbia, MO, 65211, United States of America; 3 Agriculture Experimental Station-Statistics, University of Missouri, Columbia, MO, 65211, United States of America; 4 Animal Sciences, University of Missouri, Columbia, MO, 65211, United States of America; 5 Biochemistry, University of Missouri, Columbia, MO, 65211, United States of America; 6 Genetics Area Program, University of Missouri, Columbia, MO, 65211, United States of America; University of Rennes-1, FRANCE

## Abstract

The nature and extent of care received by an infant can affect social, emotional and cognitive development, features that endure into adulthood. Here we employed the monogamous, California mouse (*Peromyscus californicus*), a species, like the human, where both parents invest in offspring care, to determine whether early exposure to endocrine disrupting chemicals (EDC: bisphenol A, BPA; ethinyl estradiol, EE) of one or both parents altered their behaviors towards their pups. Females exposed to either compound spent less time nursing, grooming and being associated with their pups than controls, although there was little consequence on their weight gain. Care of pups by males was less affected by exposure to BPA and EE, but control, non-exposed females appeared able to “sense” a male partner previously exposed to either compound and, as a consequence, reduced their own parental investment in offspring from such pairings. The data emphasize the potential vulnerability of pups born to parents that had been exposed during their own early development to EDC, and that effects on the male, although subtle, also have consequences on overall parental care due to lack of full acceptance of the male by the female partner.

## Introduction

**Fig 1:**
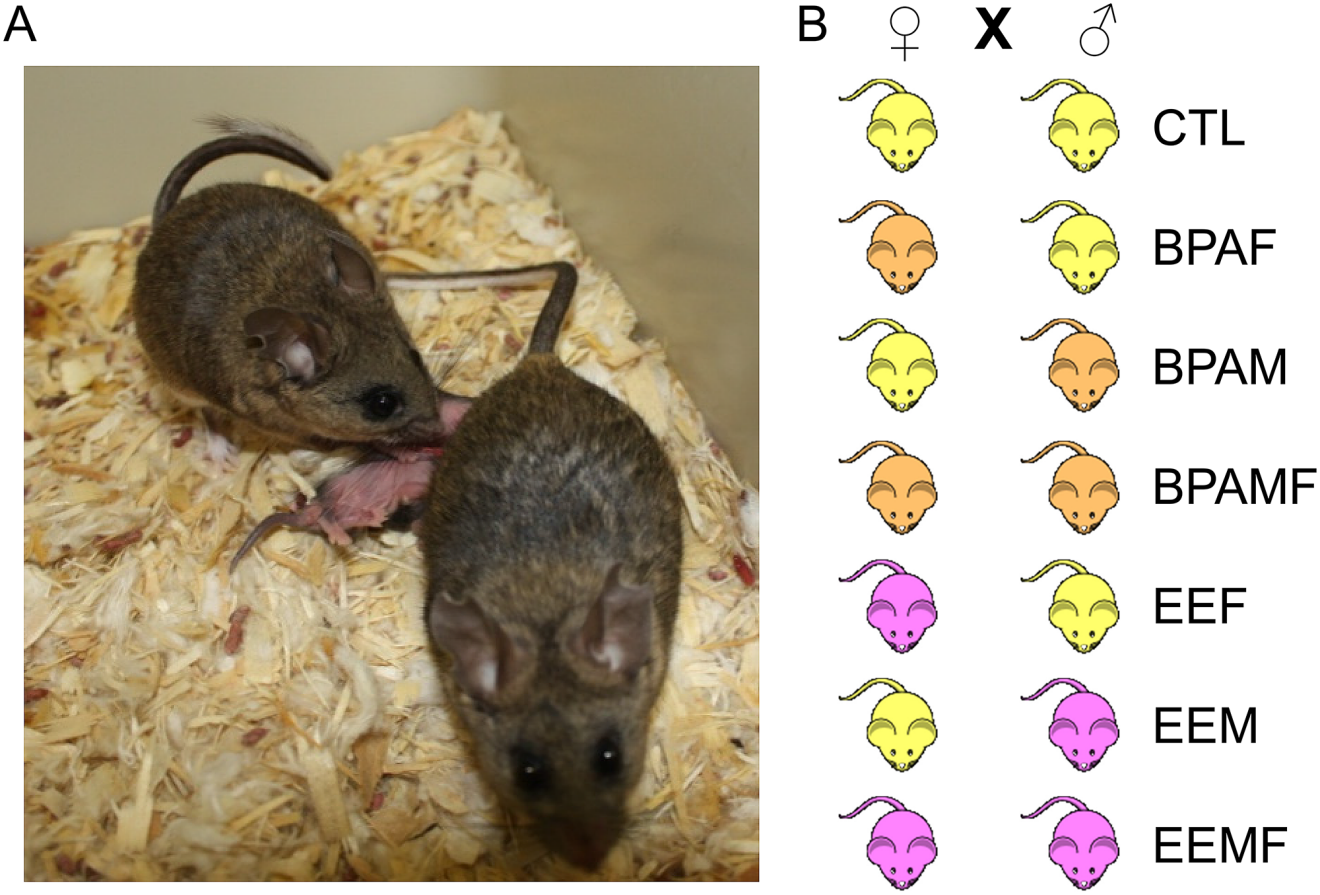
Typical image of a pair of California mice with two pups. Pups most frequently nurse from the caudally located mammary glands, while the male trails behind the female while simultaneously grooming the pups. The pairing combinations are also illustrated.

Biparental care of offspring occurs in only a minority of mammals [1] and is generally encountered in species that are socially monogamous and where the male remains bonded to the female during the period in which the offspring are conceived, suckled and ultimately weaned [2]. It occurs in primates, including humans [3], and in some rodents, but not in laboratory rats and mice, which are the most commonly used species for behavioral studies. However, paternal involvement in pup rearing has been examined in laboratory settings for only a few rodent species, including the California mouse, *Peromyscus californicus* [4–6] (Fig 1). In the latter, the male partner exhibits intense cooperative care of the pups from their birth to weaning in terms of cleaning and grooming, providing warmth by huddling over the young when the female is absent from the nest, and possibly guarding the nest and partner female from intruders. Field studies have shown that these paternal activities, some of which may be controlled by a neural circuitry homologous to that encountered in the female partner [6, 7] enhance offspring survival [8] and contribute to offspring brain development[9, 10]. Indeed impaired parental care can have dramatic epigenetic and phenotypic consequences on the young [11] and likely contributes to maladjusted social behaviors [12–14].

Several studies have shown that maternal care of offspring in mammals can be negatively affected by developmental exposure of a future female parent to extrinsic environmental factors, such as endocrine disrupting compounds that mimic the steroid hormones essential for establishing the circuitry of the adult female brain during her early development [15–20], but to our knowledge, no comprehensive analysis has been conducted on the behavior of the male partner in a species where both parents contribute to rearing the young. In the present study, we have sought to address this imbalance by exposing both male and female California mice while *in utero* and during the immediate postnatal period to the widely prevalent, industrial chemical, bisphenol A (BPA), which has estrogenic properties and known disruptive effects on maternal behaviors in laboratory mice and rats [15–20]. As an estrogen-positive control, other animals were developmentally exposed to ethinyl estradiol (EE), the main active component of birth control pills (Fig 1).

## Materials and Methods

### Animal husbandry

Outbred adult (60–90 days of age) founder California mouse females and males, free of common rodent pathogens, were purchased from the *Peromyscus* Genetic Stock Center (PGSC) at the University of South Carolina (Columbia, SC), and placed in quarantine for a minimum of 8 weeks to ensure that they did not carry any transmittable and zoonotic diseases. From the time the animals had been captured between 1979 and 1987, *P*. *californicus* captive stocks have been bred by the PGSC to maintain their outbred status. All experiments were approved by University of Missouri Animal Care and Use Committee (Protocol #7753) and performed in accordance with the recommendations in the Guide for the Care and Use of Laboratory Animals of the National Institutes of Health. Two weeks prior to breeding, virgin P_0_ females, 8 to 12 wks of age were randomly assigned to receive one of three diets: 1) a low phytoestrogen AIN 93G diet supplemented with 7% by wt corn oil to minimize potential phytoestrogenic contamination that would otherwise be present with inclusion of soybean oil in the diet, 2) this diet supplemented with 50 mg BPA/kg feed weight, which we have documented to lead to internal serum concentrations close to those measured in pregnant women unknowingly exposed to this chemical [21, 22], and 3) AIN93G diet supplemented with 0.1 parts per billion of EE, as the FDA required positive control for BPA studies [23]. The P_0_ dams remained on the diet throughout gestation and lactation, as described previously [21, 24, 25].

The F_1_ generation sons and daughters were weaned at 30 days of age. When the animals reached adulthood (~90 days of age), males and females from each groups were randomly paired (4 to 7 pairs/combination) with either controls or breeding partners developmental exposed to the same EDC such that the pairings included controls, BPA-exposed females to control males (BPAF), control females bred to BPA-exposed males (BPAM), BPA-exposed females mated to a BPA-exposed males (BPAMF), EE-exposed females mated to control males (EEF), control females bred to EE-exposed males (EEM), and EE-exposed females mated to EE-exposed males (EEFM). When the animals were not in the Phenotyper system (detailed below), they were housed in white polypropylene cages (27.8 x 7.5 x 13 cm) and maintained on a 16:8 h light: dark cycle (lights on at 6:00 A.M. CST, lights off at 10:00 P.M. CST). The EE groups were included as the Federal Drug Administration (FDA-mandated positive control for BPA studies. Additionally, EE provided insights into whether BPA effects might be due to BPA binding and activating neural ESR to the same extent as EE. We have previously validated similar effects of the chosen BPA and EE doses in a related species, deer mice (*P*. *maniculatus bairdii*) [21, 24].

Since California mice are monogamous, one male was paired with a single female, and the pair remained together for the duration of the study. California mice do not form a vaginal or copulatory plug, as observed in laboratory mice (*Mus musculus*). To determine if the females were gravid, they were weighed weekly, and five days prior to the predicted parturition date, the breeding pair was placed in the Phenotyper (Noldus Technologies, Leesburg, VA). The breeding pair and pups were kept in this cage system through five days after birth. As with the cage set-up, the animals were provided filtered water in a polypropylene water bottle. California mice typically birth about two pups in each litter, although litters sizes up to four commonly occur [26–30].

### Coding of individual, social, and parental behaviors

The Media Recorder timer program (Noldus) switched on the infra-red video-cameras to record behaviors from (MD, middle of the dark period, 1.00–2.00 h; EL, early in the light period 7.30–8.30 h; ML, middle of the light period 13.00–14.00 h; LL, late in light period, 21.00–22.00 h). To distinguish the two animals in each pair, prior to breeding, each male, under anesthesia from an intra-peritoneal (IP) injection of Avertin (250 mg/kg), was marked by an approximately 2 by 3 inch area shaved along the dorsal thoracic region. The Observer Version XI program (Noldus) was used to code the archived videos. The program allows determination of frequency and duration of specific behaviors, which were coded two days prior to birth and from post-natal day (PND) 0 (day of birth) to PND 5, as described previously [31].

### Determination of pup body weight and temperature

Beginning on PND 2, the pups were gently removed from the nest (or nipple if they were suckling), placed abdomen down on a scale (OHAUS CS200, Parsippany, NJ) that was covered with a brown paper towel, and a thermal image acquired with a FLIR i5 camera (FLIR Systems Inc., Boston, MA) with the lens 22 cm above the pup. In litters, where there was more than a single pup, individual pups on PND 2 were given a distinguishing tattoo on one of their paws on either the front or back legs (Fine Science Tools, Foster City, CA). Before the pups were returned to the nest, a thermal image of the nest area was also obtained to assess the temperature of the nest. Measurements were obtained every two days from PND 2 to 20 and then prior to and after weaning (PND 30) at 8:30, 12:30 and 16:30 h. All thermal images were analyzed by using the FLIR Tools software program (http://flir.com/tools/). Values were adjusted to represent the average temperature from the head to the base of the tail. Animals tested for this experiment include: CTL: litters = 7, pups = 15; F1 BPAF: litters = 4, pups = 6; BPAM: litters = 5, pups = 11; BPAMF: litters = 5, pups = 15; EEF: litters = 6, pups = 14; EEM: litters = 6; pups = 12; EEMF: litters = 8; pups = 16.

### Statistical analyses

#### F_1_ parental behaviors

Male and female paired behavioral data were grouped as follows: two days prior to birth, PND 0 (day of birth), PND 1–2, and PND 3–5 for each sex. For all of the PND 0 assessments, the 1:00am time point was included whether or not the pups had been born. By doing this, it was possible to allow a full rank data set for the data analysis. The male and female behaviors in a breeding pair were analyzed together and independently to determine if any pair-bond effects were evident. The behaviors that were relevant to a given sex (as detailed in [31]) were ranked due to heterogeneity of variance [32]. Parental behaviors were also analyzed by using a linear statistical model that contained the effect of sex, day (PND 0, PND 1–2, and PND 3–5), time (1:00–2:00 [MD], 7:30–8:30[EL], 13:00–14:00 [ML], and 21:00–22:00 [LL]) and all possible interactions with sex, day and time. Each breeding pair within sex was considered as the denominator of F to test sex, and pair within sex effects. Secondly, day was used as the denominator of F to test day and sex X day effects. The residual mean square of pair within sex, day, and time of day was used as the denominator of F to test time and all possible interaction of time with sex and day. The data were analyzed by using a split split plot in time analysis to account for the repeated measurements [33] and SAS version 9.2 software analyses (SAS Institute, Cary, NC). Unless otherwise stated, the reported data are based on mean ± SEM per hour assessments.

#### F_2_ sex ratio, pup weight and body temperature

F_2_ sex ratio was analyzed by using Chi-squared analysis to determine if there were differences from the expected 50:50 ratio. For the pup weight and body temperature data, two analyses were performed. Weight was analyzed as a randomized complete block design (RCBD) in which the model contained the effects of parents (combination of dam and sire), day, sex and the interaction of day X sex. The second analysis was performed on pup temperature data. The linear statistical model was a RCBD and split split plot in time. The mating pair was considered the complete block. Sex was the main plot; day and sex X day was the sub-plot; and time and all of the interactions of time with day and sex was the sub-sub-plot. All mean differences were determined by using Fisher’s Least Significance Difference (LSD). PROC MIXED procedure in SAS 9.2 was used to analyze all of the above data.

## Results

### Litter information

*P*. *californicus* females can accommodate up to four pups, but, out of 56 litters born in this and a related study, only four pairings provided four pups (7.1%), whereas 14 produced one pup (25%), and the remaining litters either two or three. Average litter size was 2.2 and did not differ between treatment groups. There was no correlation between litter size and time the mother spent nursing (P = 0.93).

### Nursing pups

**Fig 2:**
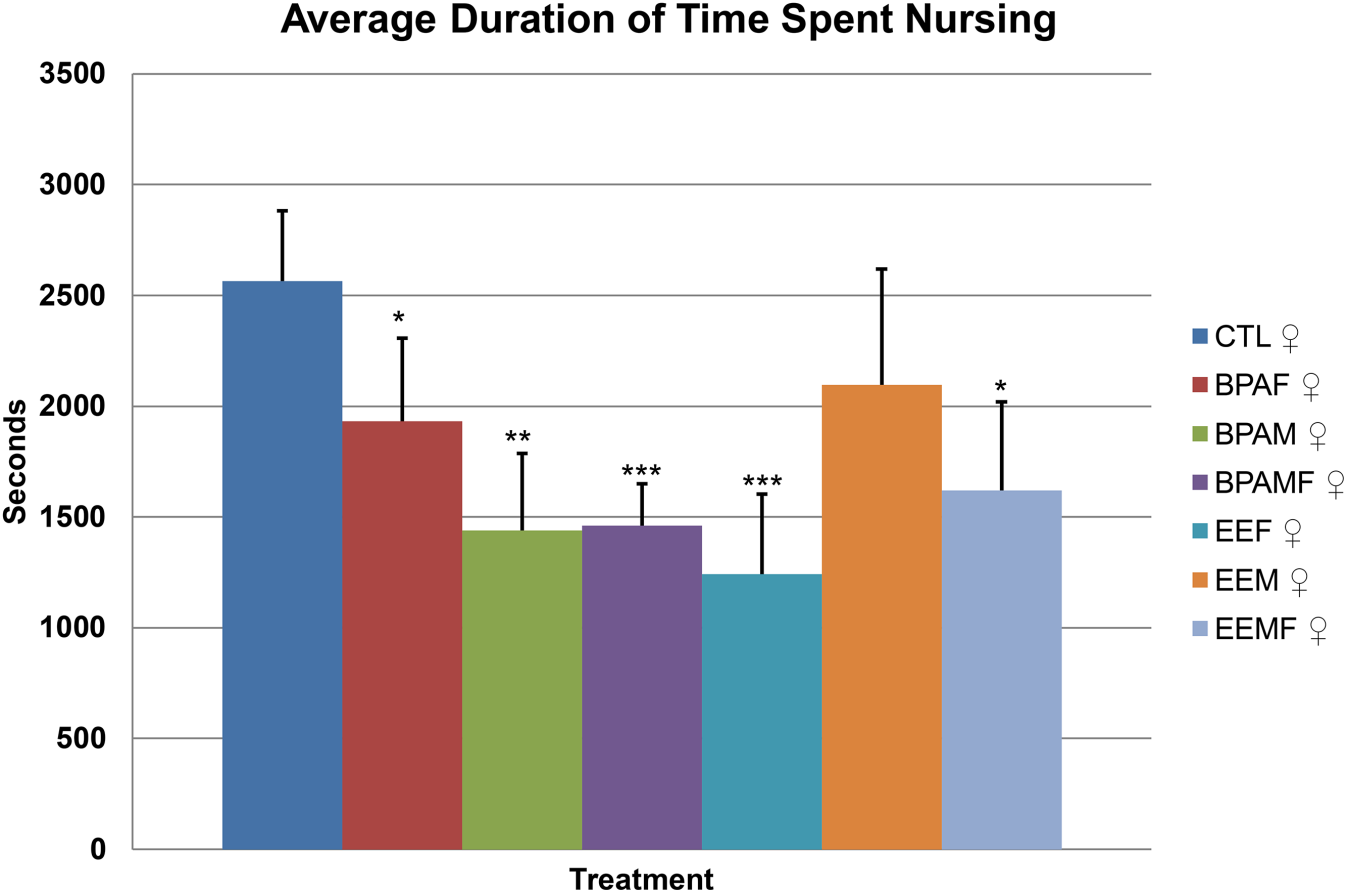
Average hourly duration of time from the day of birth to the end of post-natal day 5 that females spent nursing their pups. Treatment groups that are significantly different from control pairings are denoted with * P < 0.05; ***, P < 0.001.

Control mothers paired with a control male averaged approximately 40 min per hour with her pups attached to her nipples, and presumed to be suckling, over the period from delivery to the end of d 5 after birth (Fig 2). Mothers that had been exposed to either BPA or EE, by contrast, spent significantly less time suckling than controls over these six days. Even more surprising was that control, i.e. unexposed, females paired with BPA-exposed males suckled their young for significantly less time than controls. Moreover, this reduced suckling effect was exacerbated when both parents had been developmentally exposed. If the data were broken down into shorter time periods (S1 Fig) the same effects were observed, although the statistical power of the analyses was much reduced, particularly at post-natal day 0, where the observation period was for only 24 h and not all pairs had delivered their pups when the initial surveillance was performed at 1.00 AM. After this first day, however, the minutes a mother spent nursing within the different treatment groups did not change greatly.

### Time spent in the nest with the pups

**Fig 3:**
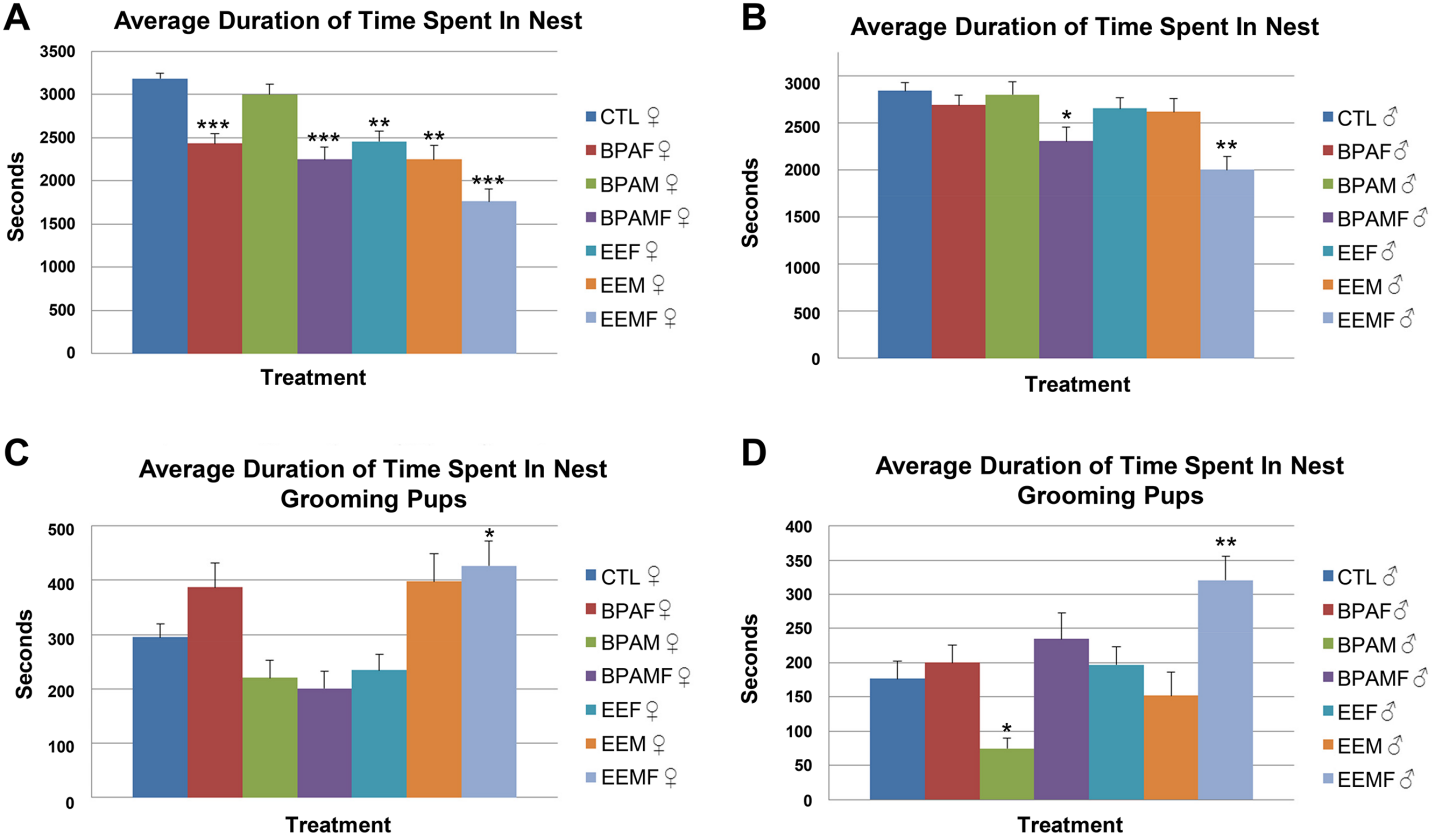
Average hourly duration of time from the day of birth to the end of post-natal day 5 that parents spent in the nest and grooming their pups. A, Time spent in nest by female parent; B, Time spent in nest by male parent; C, Time spent grooming the pups by female parent; D, Time spent grooming the pups by male parent. Treatment groups that differ from control pairs:*, P < 0.05, **, P < 0.01, and ***, P < 0.001.

BPA- and EE-exposed females spent significantly more time outside the nest and away from their pups than controls (Fig 3 and S2 Fig). Developmentally-exposed males were less affected in terms of the time they spent in association with the pups than the females. However, when both partners had been exposed, the male remained for significantly longer periods outside the nest. One possibility is that the females in these pairings actively prevented access of the males to the pups.

### Grooming pups

In *P*. *californicus*, both parents groom their pups, but the amount of time per hour they devote to this activity is quite variable (Fig 3). The prediction was that both BPA and EE pairs would engage in less grooming activities than controls, and that the effects would depend upon the sex and PND (Fig 3). In contrast to this expectation, males and females in the EEM pairings and males in the EEFM pairings groomed their pups more than observed in the controls and BPA groups. These findings suggest that the pups in these EE groups were being over-groomed rather than under-groomed.

### Effects of F_1_ parental care on F_2_ pup body weight and temperature

**Fig 4:**
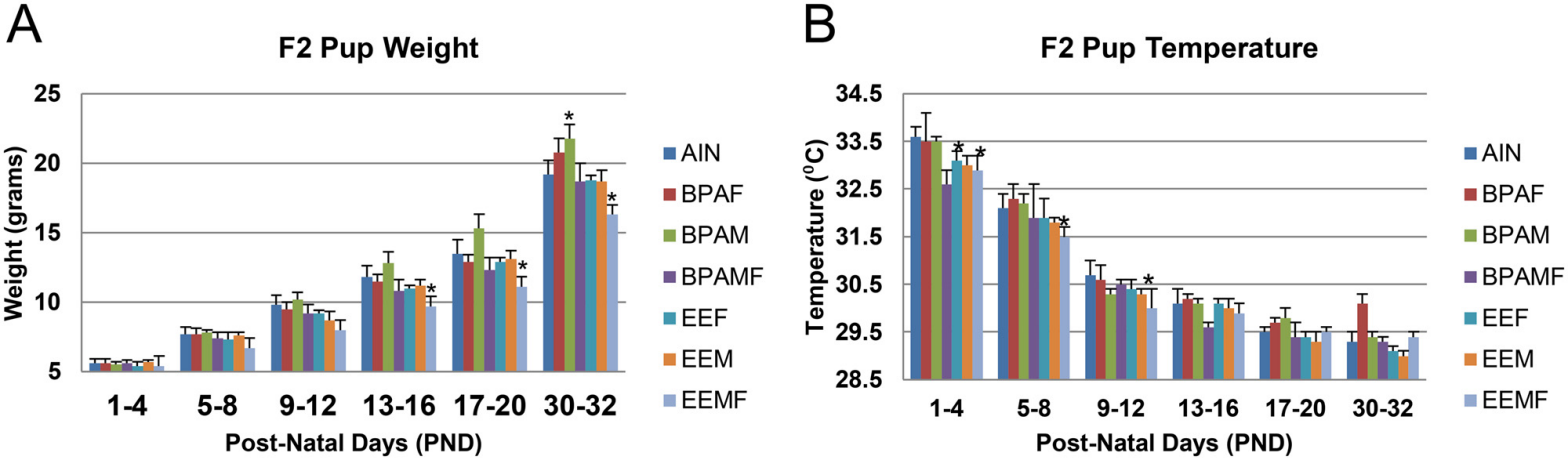
Effects of developmental EDC exposure of parents on body weight gain and body temperature of pups. A, Body weight of neonatal to weanling offspring. B, Body temperature of neonatal to weanling offspring. Treatment groups that differ from control pairs:*, P value range = 0.01 to 0.003.

Based on a Chi-squared analysis, the BPAF and BPAM group had more F_2_ sons than daughters (Table 1, P < 0.0001) but the litter number for each grouping was small and so the significance of these observations is unclear. Moreover, the BPAMF pairings provided a ratio not different than 1:1. In addition, there were no differences between the sexes for pup weight and body temperature alone and in interaction with diet (Pup weight: diet * sex, P = 0.94 and Pup body temperature: diet * sex, P = 0.09). Therefore, the results of the two sexes were combined for these categories. In most of the pairings, the pups grew at comparable rates and demonstrated similar changes in body temperature over time. The exception was F_2_ pups from the hyper-groomed EEFM pairing. These gained weight more slowly (Fig 4A) and had a lower body temperature than pups from the other pairings during the period that they rely on parental huddling, to maintain body heat [5] (Fig 4B), possibly because their parents, especially the father, spent a greater time outside the nest and away from the pups (Fig 3A & 3B). The fact that pups from BPA-exposed parents gained weight at a comparable rate to controls (Fig 4A) suggests that shorter suckling periods do not necessarily deprive pups of milk.

**Table 1:** Sex ratio of F_2_ pups from the various F_1_ treatment groups.

Treatment Group (# of litters)	Percentage of F_2_ Males (# of males)	Percentage of F_2_ Females (# of females)
**AIN (7)**	46.7 (7)	53.3 (8)
**BPAF (4)**	66.7* (4)	**33.3 (2)**
**BPAM (5)**	90.9* (10)	**9.1 (1)**
**BPAMF (5)**	53.3 (8)	46.7 (7)
**EEF (6)**	42.3 (6)	57.7 (8)
**EEM (6)**	50.0 (6)	50.0 (6)
**EEMF (8)**	43.8 (7)	56.2 (9)

*Differs significantly from expected 50:50 ratio, P < 0.0001

## Discussion

The goal of these studies was to determine how early developmental exposure to two known EDC with estrogen-like activities [34, 35], BPA and EE, might shape later maternal and paternal parenting behaviors in a monogamous, biparental mammalian species and especially how pairing with a “compromised” partner might affect parental care provided by the other partner. We also sought to determine whether or not biparental investment would be further decreased when both parents were developmentally exposed to the same environmental insult. As the brain regions and hormones regulating biparental behaviors appear to be similar across species, these studies on the California mouse may have considerable relevance to humans [36].

The experiments described here, in which it was possible to monitor the activities of both parents over several days and nights, show that early exposure of California mice to either BPA or EE at physiologically relevant concentrations [24, 37] resulted in a reduction in the amount of time a mother spent suckling her pups, greater absence from the nest by both parents, and, in the case of EE, in particular hypergrooming of the pups by both the mother and father. Accordingly, many of the health benefits ascribed to nursing, such as, cardiomyocyte development, lowered heart and metabolic rates, proper programming of mucosal and system immune systems, and eventual adult emotional and social behaviors may be blunted in F_2_ animals reared by affected dams [38, 39]. The reduced interaction with pups through suckling may also increase anxiety of the mother herself [40], and lessen oxytocin neural programming essential for strengthening the maternal-neonatal bond [41]. On the other hand, it should be noted that the reduced amount of time spent suckling did not translate into reduced growth of the pups, except in the cases where the pups were hyper-groomed. These pups also demonstrated a significantly lower body temperature than the other groups, possibly because of a combination of a less dense coat and reduced parental huddling (Fig 4). Excessive grooming may also lead to offspring epigenetic variation and later stress responses [42–44].

It is not clear why males and females in the EEM and EEMF group hyper-groomed their pups. One possibility is that thee males had become feminized in their responses, as seen in their deer mice developmentally exposed to BPA [21, 24]. Their abnormal responses may be caused by alterations in oxytocin level and ESR1 expression in the paraventricular nucleus and suproptic nuclei of the hypothalamus, as has been suggested in biparental mandarin voles (*Microtus mandarinus*) and laboratory mice (*Mus musculus*), a species that is usually regarded as predominantly maternal, with little involvement of the male in raising pups [45, 46]. The prediction at the outset was that identical effects would be observed in the BPA- and EE-exposed groups. However, this was not the case for pup grooming. As a weak estrogen, BPA may not increase oxytocin or neural ESR expression to the same extent as EE. We are currently testing these and other hypotheses to gain insights into the underlying mechanisms by which BPA and EE exposure can lead to disruptions in biparental behavior.

A handful of studies have examined the effects of acute and developmental exposure to BPA on maternal care in mice and rats (as reviewed in [47]). Depending on the dose, timing and duration of exposure, and the rodent model employed, varying effects have been reported on maternal care (see Table 1 of [47]). Prenatal exposure of F_1_ mice offspring to 10 μg BPA/kg body weight/day to the pregnant dam resulted in her daughters spending less time nursing and huddling over her pups but more time spent engaged in nest building [15]. Adult oral exposure to 40 μg BPA/kg bw/day from gestation to lactation resulted in F_0_ rat dams spending less time licking and grooming their pups and assuming an arched-back posture [17]. Developmental and adult exposure to 5 mg BPA/kg/bw/day resulted in F_1_ rats spending more time outside the nest and less time engaged in nursing [20]. F_0_ female mice exposed to 200 μg BPA/kg/bw/day engaged in less time licking, grooming, and nursing their pups [16]. Therefore, our current findings on maternal care in California mice replicate some but not all earlier findings with other rodent models. However, as well as involving different species and somewhat different experimental designs, all the prior studies employed direct oral dosing, while our study used a dietary approach to avoid stressing the mothers on a daily basis. In addition, the BPA dose we selected had been previously validated to provide similar blood concentrations of BPA to those noted in unknowingly exposed pregnant women [21, 22].

Although scant information is available about the consequences of reduced paternal care when both parents are present, effects of an absent father have been studied. Like California mice, mandarin voles (*Lasiopodomys mandarinus*) are monogamous and biparental, and pups reared solely by their mother are more anxious and exhibit poor performance in social interaction tests than offspring raised by two parents [48]. Absence of a father in California mice and in another biparental rodent, the degus (*Octodon degus)* leads to reduced dendritic and synaptic development and is accompanied by cognitive disturbances [49, 50]. Whether the reductions in biparental investment in F_2_ pups derived from BPA- and EE- exposed parents leads to similar outcomes, including lifelong neurobehavioral deficits, is unclear, but long-term studies are in process to test these possibilities., Additional studies are also needed to determine whether the altered parental care observed in the F_1_ parents is transmitted to the F_2_ generation and beyond. It is not clear why sex ratio distortions in favor of F_2_ males were evident in the BPAF and BPAM but not the BPAMF groups (Table 1). While these findings are interesting, additional studies with larger datasets are needed to confirm these findings. Sex ratio distortions can occur in individual litters, which may be the case here. In addition, they have also been observed in mothers on a high fat diet and may be caused by a variety of other factors that alter the uterine environment [51, 52].

Perhaps the most intriguing aspect of our study was that females appeared able to recognize a mate who had previously been exposed to BPA or EE. These females, although never exposed to such chemicals themselves, suckled their pups less and spent more time outside the nest than if paired with a control male. Such adjustments in maternal investment in response to perceived quality of the male partner have been reported for a number of mammalian and non-mammalian species [50, 53, 54]. However, as far as we are aware, the findings reported here are the first to demonstrate that early exposure of the male partner to an EDC, such as BPA or EE, can disturb normal patterns of care of the young by both partners and hence unleash the possibility of long term social and health consequences as their progeny mature.

## Supporting Information

S1 Fig Average duration of time spent nursing on PND 0, 1 to 2, and 3 to 5. Treatment groups that are significantly different from Controls are denoted with *, P < 0.05; **, P < 0.01.
(TIF)

S2 Fig Average duration of time spent in the nest and grooming the pups on PND 0, 1 to 2 and 3 to 5. Treatment groups that are significantly different from Controls are denoted with *, P < 0.05; **, P < 0.01.
(TIF)
