# Supplementary figures and images for: Disruption of Parenting Behaviors in California Mice, a Monogamous Rodent Species, by Endocrine Disrupting Chemicals
Source: PLoS One. 2015 Jun 3;10(6):e0126284. doi: 10.1371/journal.pone.0126284 (PMC4454565; doi:10.1371/journal.pone.0126284)

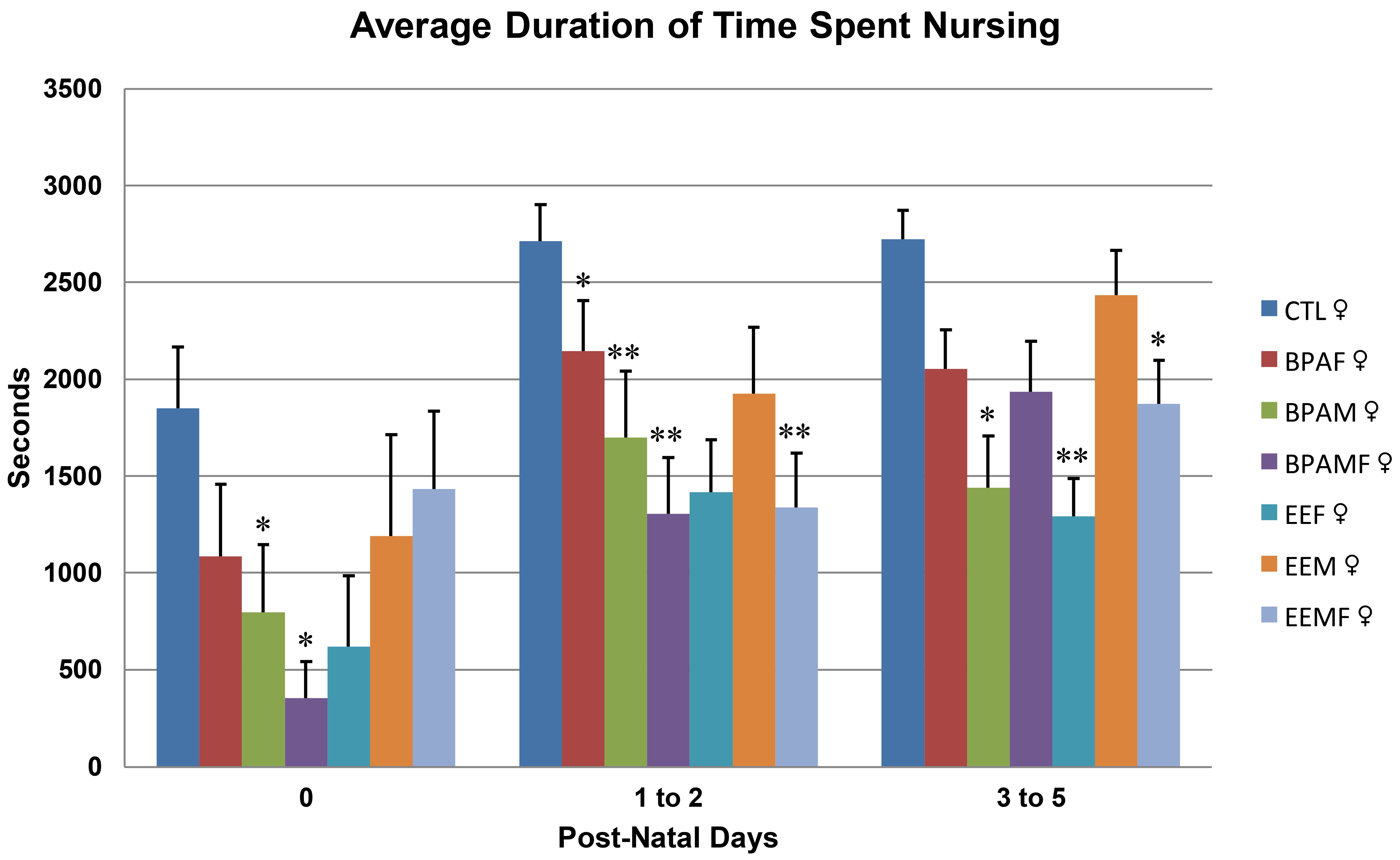

Supplement: S1 Fig — Treatment groups that are significantly different from Controls are denoted with *, P < 0.05; **, P < 0.01. (TIF) [file pone.0126284.s001.tif]

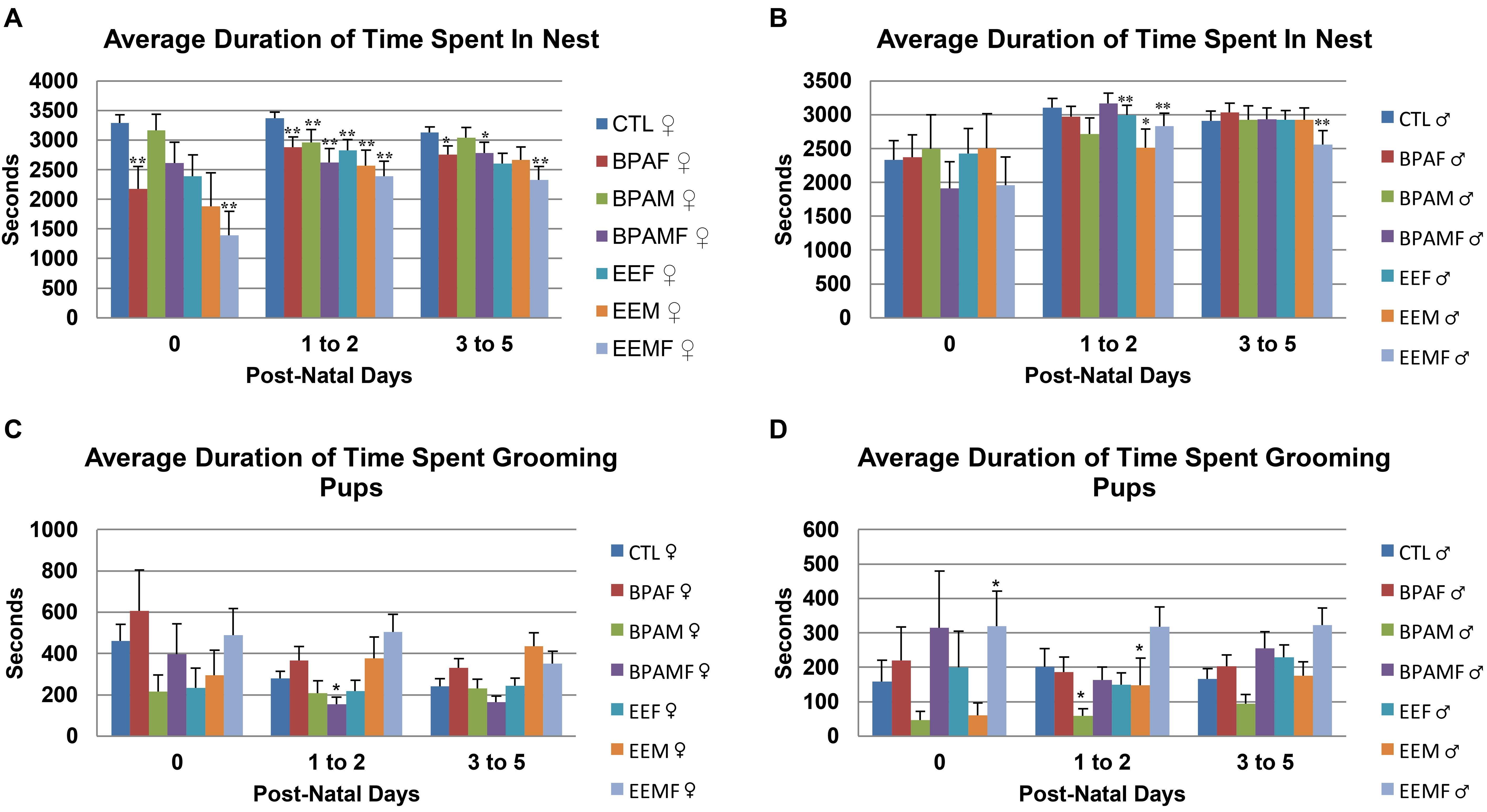

Supplement: S2 Fig — Treatment groups that are significantly different from Controls are denoted with *, P < 0.05; **, P < 0.01. (TIF) [file pone.0126284.s002.tif]
